# Genomic insights into *Lolium multiflorum* diversity for forage breeding in Andean livestock systems

**DOI:** 10.3389/fpls.2026.1818688

**Published:** 2026-06-10

**Authors:** Leidy G. Bobadilla, Rodomiro Ortiz, Gianmarco Castillo, Filipe I. Matias, Lucía Gutiérrez, Leandro Valqui, William Carrasco-Chilón, Jorge R. Díaz-Valderrama, Thiago M. Venancio, E. I. Alava, Miguel S. Castillo, Héctor V. Vásquez

**Affiliations:** 1 Escuela de Posgrado, Programa Doctoral en Ciencia para el Desarrollo Sustentable, Facultad de Ingeniería Zootecnista, Biotecnología, Agronegocios y Ciencia de Datos, Universidad Nacional Toribio Rodríguez de Mendoza de Amazonas, Chachapoyas, Chachapoyas, Peru; 2 Laboratorio de Agrostología, Instituto de Investigación en Ganadería y Biotecnología, Facultad de Ingeniería Zootecnista, Biotecnología, Agronegocios y Ciencia de Datos, Universidad Nacional Toribio Rodríguez de Mendoza de Amazonas, Chachapoyas, Chachapoyas, Peru; 3 Department of Plant Breeding, Swedish University of Agricultural Sciences (SLU), Lomma, Sweden; 4 Laboratório de Química e Função de Proteínas e Peptídeos, Centro de Biociências e Biotecnologia, Universidade Estadual do Norte Fluminense Darcy Ribeiro (LQFPP/UENF), Estado do Rio de Janeiro, Brazil; 5 Dirección de Desarrollo Tecnológico Agrario, Instituto Nacional de Innovación Agraria (INIA), Estación Experimental de Baños del Inca, Cajamarca, Peru; 6 Facultad de Ingeniería y Ciencias Agrarias, Universidad Nacional Toribio Rodríguez de Mendoza de Amazonas, Chachapoyas, Peru; 7 Facultad de Ciencias de la Vida, Escuela Superior Politécnica del Litoral (ESPOL), Guayaquil, Guayas, Ecuador; 8 Crop and Soil Sciences Department, North Carolina State University, Raleigh, NC, United States

**Keywords:** Andean grasslands, genotyping-by-sequencing, highland forage germplasm, *Lolium multiflorum*, population structure

## Abstract

Understanding the genetic diversity of *Lolium multiflorum* is essential for developing targeted breeding programs that can effectively support pasture-based livestock systems in the Peruvian Andean. This study assessed genomic variability and population structure in 27 *L. multiflorum* accessions from the Cajamarca region and the INIA Amazonas germplasm bank (Peru), using the genotyping-by-sequencing (GBS) technique. DNA extracted from young leaves was sequenced on an Illumina NovaSeq 6000 platform. After bioinformatic processing, 2,070 single nucleotide polymorphisms (SNPs) with heterogeneous distribution were obtained across seven chromosomes. A Principal Coordinate and an Unweighted Pair Group Method with Arithmetic Mean analyses revealed two distinct genetic groups were identified, reflecting a complex structure shaped by gene flow and local selection. The analysis of molecular variance showed that 90% of the genetic variation occurs within populations, whereas the remaining 10% corresponds to interregional differences (PhiPT = 0.099, p < 0.006). The negative Inbreeding Coefficient (F_IS_) values (Cajamarca = -0.2312; Amazonas = -0.5489) indicate an excess of heterozygotes, a pattern typically associated with predominantly outcrossing species. Additionally, high observed heterozygosity (Ho > 0.57) points to potential hybrid vigor and indicates that these populations may maintain stable genetic equilibrium. Collectively, these findings demonstrate that Peruvian *L. multiflorum* harbors a broad genetic base, shaped by historical germplasm exchange and local environmental adaptation. This diversity provides critical insights for conservation strategies and can supports breeding programs aimed at enhancing forage resilience and productivity in high-Andean ecosystems.

## 1 Introduction

Grasslands cover more than 40% of the Earth’s ice-free land surface and provide essential ecosystem services, including livestock products, carbon storage, and nutrient cycling (Millennium Ecosystem Assessment, 2005; Hewins et al., 2018; Erb et al., 2016). Land-use intensification and inadequate management practices negatively affect grassland ecosystems, resulting in biomass loss, biodiversity decline, and reduced ecosystem functioning (Chang et al., 2020; Jiao et al., 2024; Cao et al., 2024). Mountain grasslands are particularly vulnerable to these pressures, as pronounced environmental gradients and climatic variability can accelerate degradation processes and constrain the resilience of pasture-based livestock systems (Rui et al., 2025). In this context, conserving and characterizing genetic diversity of forage species is critical for sustaining productive and resilient livestock systems.

*Lolium multiflorum* Lam. is a widely cultivated forage species in temperate and high-altitude regions due to its high herbage mass production, nutritional value, and capacity to perform under diverse environmental conditions (Carrasco-Chilón et al., 2024). As a self-incompatible, obligate outcrossing species, *L. multiflorum* typically exhibits high levels of genetic variation within populations, providing a solid foundation for selection and genetic improvement (Fernández-Otero et al., 2024). Studies across the genus *Lolium* have consistently reported that most genetic variation occurs within populations rather than among them, emphasizing the importance of local germplasm for breeding and conservation programs (Nam et al., 2025).

Despite its agronomic relevance in Andean livestock systems, the genomic diversity and population structure of *L. multiflorum* in the Peruvian Andes remain poorly characterized (Vallejos-Cacho et al., 2024). Most genomic research on ryegrass has focused on European and Asian germplasm (Johansen et al., 2025), whereas genomic information for Andean populations remains limited. In the Peruvian Andes, *L. multiflorum* has been cultivated under heterogeneous environmental conditions and diverse management practices, which may contribute to genetic differentiation and local adaptation. In the Peruvian Andes, *L. multiflorum* is cultivated between 2,300 and 3,800 m.a.s.l. under highly heterogeneous environmental conditions associated with altitudinal gradients (Vallejos-Fernández et al., 2020). Traditional management practices, including informal seed exchange, intensive sowing, and recurrent reseeding, combined with an allogamous mating system, are expected to influence patterns of genetic diversity and population structure (Vieira et al., 2004). These conditions make Andean germplasm particularly well suited for genome-wide diversity analyses.

Single-nucleotide polymorphism (SNP) markers generated through genotyping-by-sequencing (GBS) provide an efficient and reproducible approach for assessing genomic diversity in forage species (Elshire et al., 2011; Jaškūnė et al., 2020; Altaf et al., 2025). SNP datasets derived from GBS have been widely applied to infer genetic diversity, population structure, and genetic relationships in *Lolium* species and other forage crops (Blackmore et al., 2016; Tamura et al., 2022; Altaf et al., 2025). The application of SNP- and GBS-based approaches to Andean ryegrass germplasm addresses an important knowledge gap regarding the genomic organization of Peruvian populations and contributes to a more complete understanding of forage biodiversity in high-altitude environments (Kumar et al., 2024; Bunjkar et al., 2024). Hence, the objective of this study was to characterize, at the genomic scale, the genetic diversity and population structure of *L. multiflorum* accessions from the northern Peruvian Andes using SNP-based GBS with the goals to identify a genomic baseline for Peruvian ryegrass germplasm and to offer a scientific foundation to guide forage breeding programs, genetic conservation strategies, and the adaptation of Andean livestock systems to high-altitude environmental variability.

## 2 Materials and methods

### 2.1 Plant material

A total of 50 ryegrass accession samples were collected in 2025 from selected accessions maintained in the germplasm bank of the National Institute for Agricultural Innovation (INIA) in Peru, at geographic coordinates 78°27′07″ W longitude and 07°09′56″ S latitude, at an elevation of 2667 m a.s.l. These samples represented the complete ryegrass germplasm collection conserved by INIA, maintained *in situ* and properly managed to prevent crossing among accessions. Sampling was conducted randomly within each plot; plant material was obtained from multiple non-adjacent individuals, maintaining a minimum distance of 5 m between plants. The collected accessions originated from the Cajamarca Region at the following sites: Cutervo (5), Tacabamba (7), Sendamal (5), Paccha (4), El Agrario (4), Calquís (4), Bambamarca (4), Campiña (3), Santa Cruz (2), San Pablo (1), Cochan (3), Celendín (4), and Baños del Inca (4).

**Figure 1:**
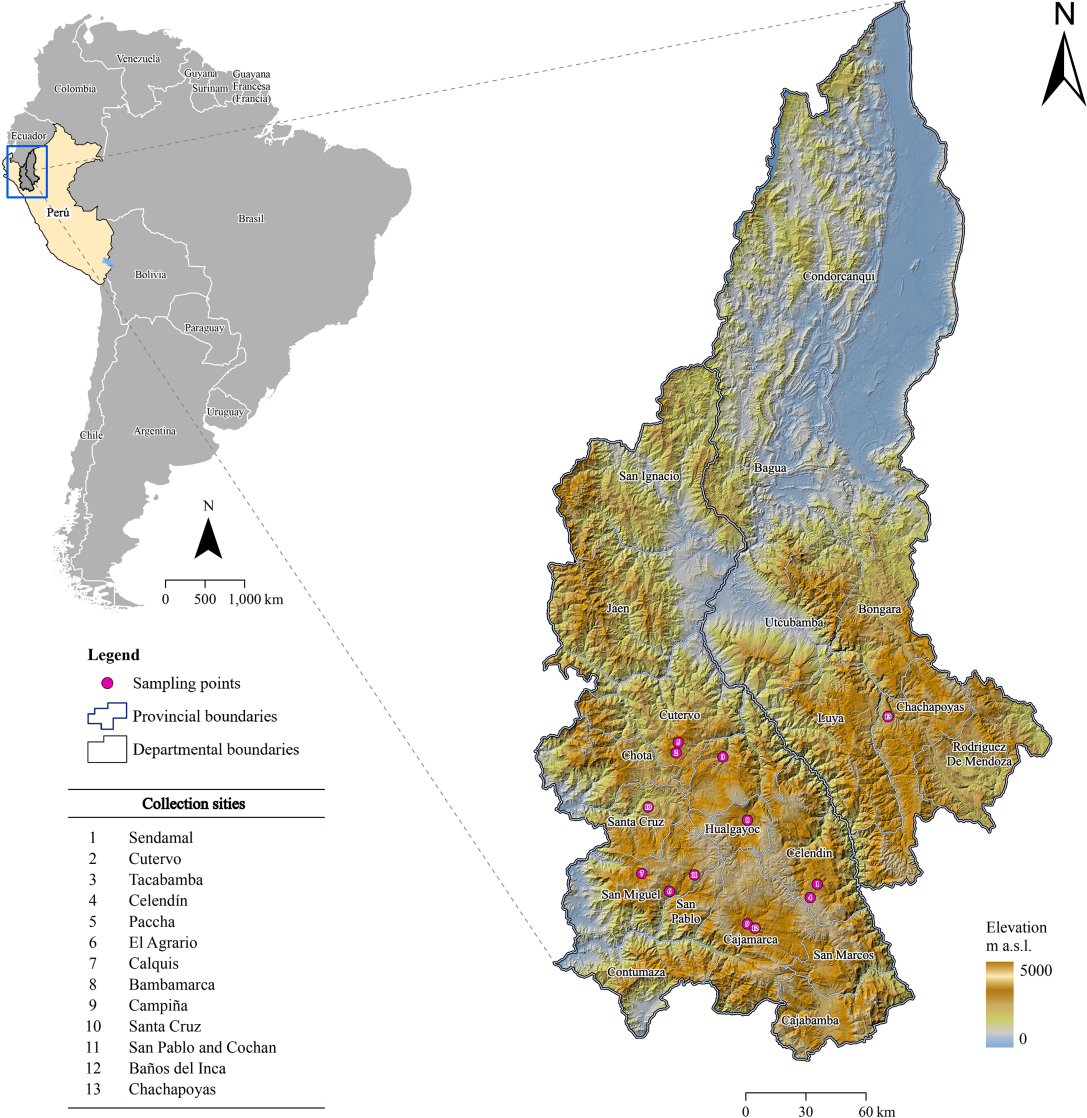
Collecting sites of *L. multiflorum* accessions.

In addition, 12 commercial ryegrass accessions were collected from materials available at the Chachapoyas Agricultural Experiment Station, within the Agrostological Garden (institutional forage collection) of the Universidad Nacional Toribio Rodríguez de Mendoza de Amazonas, Amazonas Region. These included the following cultivars: Wanca Grass (3), Bison II (3), Inglés (2), Max (2), and the AGP ecotype (2) (**Figure 1**; **Table 1**).

**Table 1:** Identification and origin of *L. multiflorum* accessions.

Sample ID	Accession name	Collection site	Province	Department	Coordinates	
					East	North
LM1 - P1	*Lolium multiflorum Paccha - LM1*	Paccha	Chota	Cajamarca	78°48′40.85″ W	6°19′43.16″ S
LM1 - P2	*Lolium multiflorum Paccha - LM1*	Paccha	Chota	Cajamarca	78°48′40.85″ W	6°19′43.16″ S
LM1 - P3	*Lolium multiflorum Paccha - LM1*	Paccha	Chota	Cajamarca	78°48′40.85″ W	6°19′43.16″ S
LM1 - P4	*Lolium multiflorum Paccha - LM1*	Paccha	Chota	Cajamarca	78°48′40.85″ W	6°19′43.16″ S
LM2 - P1	*Lolium multiflorum Cutervo - LM2*	Cutervo	Cutervo	Cajamarca	78°49′15.76″ W	6°22′34.56″ S
LM2 - P2	*Lolium multiflorum Cutervo - LM2*	Cutervo	Cutervo	Cajamarca	78°49′15.76″ W	6°22′34.56″ S
LM2 - P3	*Lolium multiflorum Cutervo - LM2*	Cutervo	Cutervo	Cajamarca	78°49′15.76″ W	6°22′34.56″ S
LM2 - P4	*Lolium multiflorum Cutervo - LM2*	Cutervo	Cutervo	Cajamarca	78°49′15.76″ W	6°22′34.56″ S
LM3 - P1	*Lolium multiflorum Tacabamba - LM3*	Tacabamba	Chota	Cajamarca	78°36′36.29″ W	6°23′37.05″ S
LM3 - P2	*Lolium multiflorum Tacabamba - LM3*	Tacabamba	Chota	Cajamarca	78°36′36.29″ W	6°23′37.05″ S
LM3 - P3	*Lolium multiflorum Tacabamba - LM3*	Tacabamba	Chota	Cajamarca	78°36′36.29″ W	6°23′37.05″ S
LM3 - P4	*Lolium multiflorum Tacabamba - LM3*	Tacabamba	Chota	Cajamarca	78°36′36.29″ W	6°23′37.05″ S
LM4 - P1	*Lolium multiflorum Tacabamba- LM4*	Tacabamba	Chota	Cajamarca	78°36′36.29″ W	6°23′37.05″ S
LM4 - P2	*Lolium multiflorum Tacabamba- LM4*	Tacabamba	Chota	Cajamarca	78°36′36.29″ W	6°23′37.05″ S
LM4 - P3	*Lolium multiflorum Tacabamba- LM4*	Tacabamba	Chota	Cajamarca	78°36′36.29″ W	6°23′37.05″ S
LM5 - P1	*Lolium multiflorum Cutervo- LM5*	Cutervo	Cutervo	Cajamarca	78°49′15.76″ W	6°22′34.56″ S
LM6 - P1	*Lolium multiflorum Calquis - LM6*	Calquis	San Miguel	Cajamarca	78°58′26.35″ W	6°55′15.54″ S
LM6 - P2	*Lolium multiflorum Calquis - LM6*	Calquis	San Miguel	Cajamarca	78°58′26.35″ W	6°55′15.54″ S
LM6 - P3	*Lolium multiflorum Calquis - LM6*	Calquis	San Miguel	Cajamarca	78°58′26.35″ W	6°55′15.54″ S
LM6 - P4	*Lolium multiflorum Calquis - LM6*	Calquis	San Miguel	Cajamarca	78°58′26.35″ W	6°55′15.54″ S
LM7 - P1	*Lolium multiflorum El Agrario - LM7*	El Agrario	San Miguel	Cajamarca	78°50′55.37″ W	7°0′2.52″ S
LM7 - P2	*Lolium multiflorum El Agrario - LM7*	El Agrario	San Miguel	Cajamarca	78°50′55.37″ W	7°0′2.52″ S
LM7 - P3	*Lolium multiflorum El Agrario - LM7*	El Agrario	San Miguel	Cajamarca	78°50′55.37″ W	7°0′2.52″ S
LM7 - P4	*Lolium multiflorum El Agrario - LM7*	El Agrario	San Miguel	Cajamarca	78°50′55.37″ W	7°0′2.52″ S
LM8 - P1	*Lolium multiflorum Bambamarca - LM8*	Bambamarca	Hualgayoc	Cajamarca	78°29′52.3″ W	6°40′42.1″ S
LM8 - P2	*Lolium multiflorum Bambamarca - LM8*	Bambamarca	Hualgayoc	Cajamarca	78°29′52.3″ W	6°40′42.1″ S
LM8 - P3	*Lolium multiflorum Bambamarca - LM8*	Bambamarca	Hualgayoc	Cajamarca	78°29′52.3″ W	6°40′42.1″ S
LM8 - P4	*Lolium multiflorum Bambamarca - LM8*	Bambamarca	Hualgayoc	Cajamarca	78°29′52.3″ W	6°40′42.1″ S
LM9 - P1	*Lolium multiflorum Campiña- LM9*	Campiña	Cajamarca	Cajamarca	78°29′46.61″ W	7°8′46.53″ S
LM10 - P1	*Lolium multiflorum Campiña- LM10*	Campiña	Cajamarca	Cajamarca	78°29′46.61″ W	7°8′46.53″ S
LM11 - P1	*Lolium multiflorum Sendamal- LM11*	Sendamal	Celendín	Cajamarca	78°10′52.49″ W	6°57′54.96″ S
LM11 - P2	*Lolium multiflorum Sendamal- LM11*	Sendamal	Celendín	Cajamarca	78°10′52.49″ W	6°57′54.96″ S
LM12 - P1	*Lolium multiflorum Sendamal- LM12*	Sendamal	Celendín	Cajamarca	78°10′52.49″ W	6°57′54.96″ S
LM12 - P2	*Lolium multiflorum Sendamal- LM12*	Sendamal	Celendín	Cajamarca	78°10′52.49″ W	6°57′54.96″ S
LM12 -P3	*Lolium multiflorum Sendamal- LM12*	Sendamal	Celendín	Cajamarca	78°10′52.49″ W	6°57′54.96″ S
LM13 - P1	*Lolium multiflorum Celendín- LM13*	Celendín	Cajamarca	Cajamarca	78°12′45.07″ W	7°1′32.91″ S
LM13 - P2	*Lolium multiflorum Celendín- LM13*	Celendín	Cajamarca	Cajamarca	78°12′45.07″ W	7°1′32.91″ S
LM13 - P3	*Lolium multiflorum Celendín- LM13*	Celendín	Cajamarca	Cajamarca	78°12′45.07″ W	7°1′32.91″ S
LM13 - P4	*Lolium multiflorum Celendín- LM13*	Celendín	Cajamarca	Cajamarca	78°12′45.07″ W	7°1′32.91″ S
LM14 - P1	*Lolium multiflorum Campiña- LM14*	Campiña	Cajamarca	Cajamarca	78°29′46.61″ W	7°8′46.53″ S
LM15 - P1	*Lolium multiflorum Cochan- LM15*	Cochan	San Pablo	Cajamarca	78°44′9.65″ W	6°55′34.67″ S
LM16 - P1	*Lolium multiflorum Cochan- LM16*	Cochan	San Pablo	Cajamarca	78°44′9.65″ W	6°55′34.67″ S
LM17 - P1	*Lolium multiflorum Cochan- LM17*	Cochan	San Pablo	Cajamarca	78°44′9.65″ W	6°55′34.67″ S
LM18 - P1	*Lolium multiflorum Santa Cruz- LM18*	Santa Cruz	Santa Cruz	Cajamarca	78°56′45.22″ W	6°37′15.11″ S
LM19 - P1	*Lolium multiflorum Santa Cruz- LM19*	Santa Cruz	Santa Cruz	Cajamarca	78°56′45.22″ W	6°37′15.11″ S
LM20 - P1	*Lolium multiflorum San Pablo - LM20*	San Pablo	San Pablo	Cajamarca	78°44′9.65″ W	6°55′34.67″ S
LM21 - P1	*INIA 910 – Kumymarca- LM21*	Baños del Inca	Cajamarca	Cajamarca	78°27′39.55″ W	7°9′53.78″ S
LM21 - P2	*INIA 910 – Kumymarca- LM21*	Baños del Inca	Cajamarca	Cajamarca	78°27′39.55″ W	7°9′53.78″ S
LM21 - P3	*INIA 910 – Kumymarca- LM21*	Baños del Inca	Cajamarca	Cajamarca	78°27′39.55″ W	7°9′53.78″ S
WC - P1	Wanca grass-Comercial	Comercial	Chachapoyas	Amazonas	77°52′0.75″ W	6°12′27.03″ S
WC - P2	Wanca grass-Comercial	Comercial	Chachapoyas	Amazonas	77°52′0.75″ W	6°12′27.03″ S
WC- P3	Wanca grass-Comercial	Comercial	Chachapoyas	Amazonas	77°52′0.75″ W	6°12′27.03″ S
BISII - P1	Bison II-Comercial	Comercial	Chachapoyas	Amazonas	77°52′0.75″ W	6°12′27.03″ S
BISII - P2	Bison II-Comercial	Comercial	Chachapoyas	Amazonas	77°52′0.75″ W	6°12′27.03″ S
BISII - P3	Bison II-Comercial	Comercial	Chachapoyas	Amazonas	77°52′0.75″ W	6°12′27.03″ S
Ingl - P1	Ingles-Comercial	Comercial	Chachapoyas	Amazonas	77°52′0.75″ W	6°12′27.03″ S
Ingl - P2	Ingles-Comercial	Comercial	Chachapoyas	Amazonas	77°52′0.75″ W	6°12′27.03″ S
MAX - P1	Max-Comercial	Comercial	Chachapoyas	Amazonas	77°52′0.75″ W	6°12′27.03″ S
MAX – P3	Max-Comercial	Comercial	Chachapoyas	Amazonas	77°52′0.75″ W	6°12′27.03″ S
AGP - P1	Ecotipo AGP-Comercial	Comercial	Chachapoyas	Amazonas	77°52′0.75″ W	6°12′27.03″ S
AGP - P2	Ecotipo AGP-Comercial	Comercial	Chachapoyas	Amazonas	77°52′0.75″ W	6°12′27.03″ S
LM 22 - P1	Ecotipo Cajamarquino *LM-22*	Baños del Inca	Cajamarca	Cajamarca	78°27′39.55″ W	7°9′53.78″ S

A total of 62 samples were collected. Samples corresponding to repeated collections of the same accession were concatenated and analyzed as a single representative accession, resulting in a final dataset of 27 accessions used in the genetic analyses.

### 2.2 DNA extraction

Ten young leaves were collected from each ryegrass accession and stored in Ziploc bags with silica gel to preserve the plant material prior to DNA extraction (Wilkie et al., 2013). Each ryegrass accession was then cut until a total weight of 650 mg per accession was obtained. The samples were transferred to 1.5-mL microcentrifuge tubes and mechanically disrupted using a tissue homogenizer. Grinding was performed using liquid nitrogen to ensure efficient homogenization. DNA extraction was carried out using the NucleoSpin^®^ Plant II kit (MACHEREY-NAGEL, Dueren, Germany) following the manufacturer’s instructions for grasses. Upon completion of the extraction process, the quality of the extracted DNA was assessed using the Quantus Fluorometer (Promega, Madison, USA). DNA integrity was verified through 1% agarose gel electrophoresis using a 1X Tris-Acetate-EDTA (TAE) buffer, and GelRed (Merck, Rahway, New Jersey, USA) for loading. The DNA samples were further stored at -20 °C for preservation. The material was later sent to Novogene (Sacramento, CA, United States) for high-throughput sequencing.

### 2.3 Sequencing and SNP genotyping analysis

Sequencing-based genotyping libraries were developed following the protocol of Elshire et al. (2011). Genomic DNA was digested with the ApeKI enzyme, and fragments were ligated to Illumina sequencing adapters and sequence barcodes unique to each sample. After multiplexing, this allowed for the recovery of sample identity for each sequenced DNA fragment. Pooled samples were sequenced on the Illumina NovaSeq 6000 platform, obtaining 100-bp paired-end reads. Raw data quality was examined using FastQC v0.11.726. The reads obtained from the 62 ryegrass samples were filtered into clean data using Fastp v0.22.0 (Chen et al., 2018). The BWA v0.7.18 software (Li and Durbin, 2009) was used to align the reads to the *L. multiflorum* genome (GCA_030979885.1) of the Rabiosa cultivar (Goettelmann et al., 2024). The alignment files were processed using SAMTOOLS v1.22.1 (Li et al., 2009), reorganized and corrected with samtools fixmate, and reweighted with PICARD v3.3.0 (https://github.com/broadinstitute/picard). SNPs within the 62 sequences of the ryegrass accessions were called using GATK v4.6.2 (Auwera et al., 2013). Filtering was performed using the following parameters: SNPs were selected while excluding indels and other variants; only biallelic SNPs were retained; QD < 2.0, FS > 60.0, MQ < 40.0, MQRankSum < –12.5, ReadPosRankSum < –8.0, QUAL < 30.0, and 10 ≤ DP ≤ 100 (Song et al., 2023). Additionally, the VCF files generated with GATK v4.6.2 were concatenated using BCFtools v1.22 (Danecek and McCarthy, 2017).

The initial dataset consisted of 62 ryegrass plant samples. However, some of these samples were biological replicates of the same genetic accession. Therefore, the number of samples was not equivalent to the number of unique accessions. To resolve this, replicate samples were merged by retaining a single representative genotype per accession, thereby removing redundancy. This procedure generated a final non-redundant dataset of 27 unique accessions, which were used for downstream genomic analyses (**Table 1**). This approach was implemented to avoid redundancy and pseudo-replication, ensuring that downstream population genetic analyses reflected true genetic diversity rather than replicate structure. Subsequently, the data were curated using VCFtools v0.1.17 (Danecek et al., 2011) by applying filters for MAF > 0.1, missing data ≤ 0.1, and MAC ≥ 3 (Arbizu et al., 2025). Finally, SNPs in linkage disequilibrium (Ragsdale and Gravel, 2020) with (r² = 0.85) were removed using cyvcf2 v0.31.2 (Pedersen and Quinlan, 2017) and scikit-allel v1.3.13 (Miles et al, 2024) in Python v3.12.3. This computational approach enabled the retrieval of highly curated SNPs and a robust inference of the genetic relationships among the accessions.

### 2.4 Analysis of population structure and genetic diversity

Population genetic structure was analyzed using ADMIXTURE v1.3.0 (Alexander and Lange, 2011) with the maximum-likelihood algorithm. Values of K from 1 to 10 were evaluated, performing 10 independent replicates for each K, and cross-validation was used to estimate the CV error and determine the optimal number of clusters. For each value of K, the replicate with the highest likelihood reported by the software was selected for subsequent analyses. Population structure was visualized using the ‘ggplot2’ package v4.0.0 (Wickham, 2016) in R. Additionally, to determine the ancestry of the accessions within each ADMIXTURE cluster, the Weir and Cockerham (1984) FST was calculated. For this analysis, the packages *hierfstat* v0.5.11 (Goudet, 2005) and *adegenet* v2.1.11 (Jombart, 2008) were used. Likewise, an individual-by-individual genetic correlation matrix was generated by calculating the identity-by-state coefficient (PI_HAT). For this calculation, the filtered and robust SNPs obtained and PLINK v1.9.0 (Purcell et al., 2007) software were used to process the data and generate the similarity matrix. Visualization was performed using the pheatmap v1.0.12 (Kolde, 2019) package in R.

A molecular analysis of variance (AMOVA) was also performed using the R ‘ape’ package (Paradis and Schliep, 2019) to determine the sources of genetic variance among ryegrass lineages. To evaluate statistical significance, a randomization test with 999 permutations was conducted using the permutation test function of the ade4 package v1.7.23 (Dray and Dufour, 2007). Subsequently, genetic diversity parameters were estimated, including sample size (N), observed heterozygosity (Ho), expected heterozygosity (He), inbreeding coefficient (F_IS_), and the Shannon diversity index, using the Hierfstat v0.5.11 package (Goudet, 2005) in R. Levels of genetic diversity were classified following Kanaka et al. (2023) and Mohammadi and Prasanna (2003), considering expected heterozygosity (He) values close to one as indicative of a high number of alleles with similar frequencies, and values close to zero as indicative of no heterozygosity.

Principal Coordinate Analysis (PCoA) was performed in R. Genotypes were extracted from the previously filtered VCF file using the ‘vcfR’ package v.15.0 (Knaus and Grünwald, 2017). Euclidean distances among individuals were computed using ‘ape’ v5.8.1 (Paradis and Schliep, 2019), and principal coordinates were obtained through the PCoA function of the same package. Visualization of the results was carried out with ‘ggplot2’, and marginal distributions were added using ggExtra v0.11.0 (Attali and Baker, 2019) to represent both intra- and interregional genetic dispersion and variability.

Additionally, a principal component analysis (PCA) was performed using productive traits related to dry matter (DM) and fresh forage (FF) to explore phenotypic patterns among the accessions. This analysis was conducted on the final set of 27 accessions and is presented as supplementary material (**Supplementary Figure 3**).

### 2.5 Phylogenetic analysis

The phylogenetic tree was constructed using the Unweighted Pair Group Method with Arithmetic Mean (UPGMA) based on the total high-quality SNPs obtained, calculating binary genetic distances through Hamming distance in Python v3.12.3, using the NumPy (Harris et al., 2020), SciPy v1.15.3 (Virtanen et al., 2020), scikit-allel 1.3.13 (Miles et al., 2024), and Bio.Phylo v1.85 (Talevich et al., 2012) packages. Hamming distance was selected because it efficiently measures pairwise dissimilarity among binary SNP genotypes by counting allelic mismatches between individuals (Wang et al., 2015; Agazzi et al., 2023; Sanjeevan and König, 2025). This metric is commonly applied in SNP-based phylogenetic and population analyses, as it preserves marker-level differences and is computationally efficient, making it suitable even when large volumes of genomic data are not available (Hamming, 1950; Di Pasquale et al., 2021). Biallelic genotypes were generated with scikit-allel, and the robustness of the clustering was evaluated using 1,000 bootstrap replicates by resampling loci. The tree was exported in Newick format and visualized in iTOL v7 (Letunic and Bork, 2024). This procedure ensured a reproducible and consistent phylogenetic inference based on high-quality SNP data.

## 3 Results

### 3.1 Sequencing and distribution of SNPs

The total raw sequencing reads for the 27 *L. multiflorum* accessions were 320,554,538, with an average of 11.9 million reads per accession. Variant calling performed with GATK identified a total of 7,379,574 SNPs across the 27 accessions, which were then filtered to ensure the quality and reliability of the analyzed variants. After applying the GATK filtering criteria, 614,161 high-quality variants were retained. Subsequently, 22,689 SNPs were removed using VCFtools, and a final linkage disequilibrium (LD) filtering step implemented with the cyvcf2 and scikit-allel packages yielded a curated set of 2,070 high-quality SNPs distributed across the seven ryegrass chromosomes (**Table 2**). This rigorous filtering strategy ensured a robust and representative marker dataset suitable for downstream genomic analyses. The highest number of SNPs was physically mapped to chromosome four (19.28%, 399 SNPs), and the lowest to chromosome six (10.68%, 221 SNPs). Chromosomes 1 (1.18 Mb) and 7 (0.77 Mb) showed the highest and lowest SNP densities, respectively.

**Table 2:** Genomic distribution of 2,070 single nucleotide polymorphisms (SNPs) across the seven ryegrass chromosomes.

Chromosome	SNP (#)	SNP (%)	Total length (Mb)	Average total (Pb)	Density (SNPs/Mb)
1	303	14.64	257.70	853314.15	1.18
2	350	16.91	341.23	977724.86	1.03
3	315	15.22	355.29	1131510.51	0.89
4	399	19.28	425.99	1070333.75	0.94
5	223	10.77	242.22	1091079.00	0.92
6	221	10.68	254.32	1156006.58	0.92
7	259	12.51	334.89	1298019.17	0.77

### 3.2 Population structure

**Figure 2:**
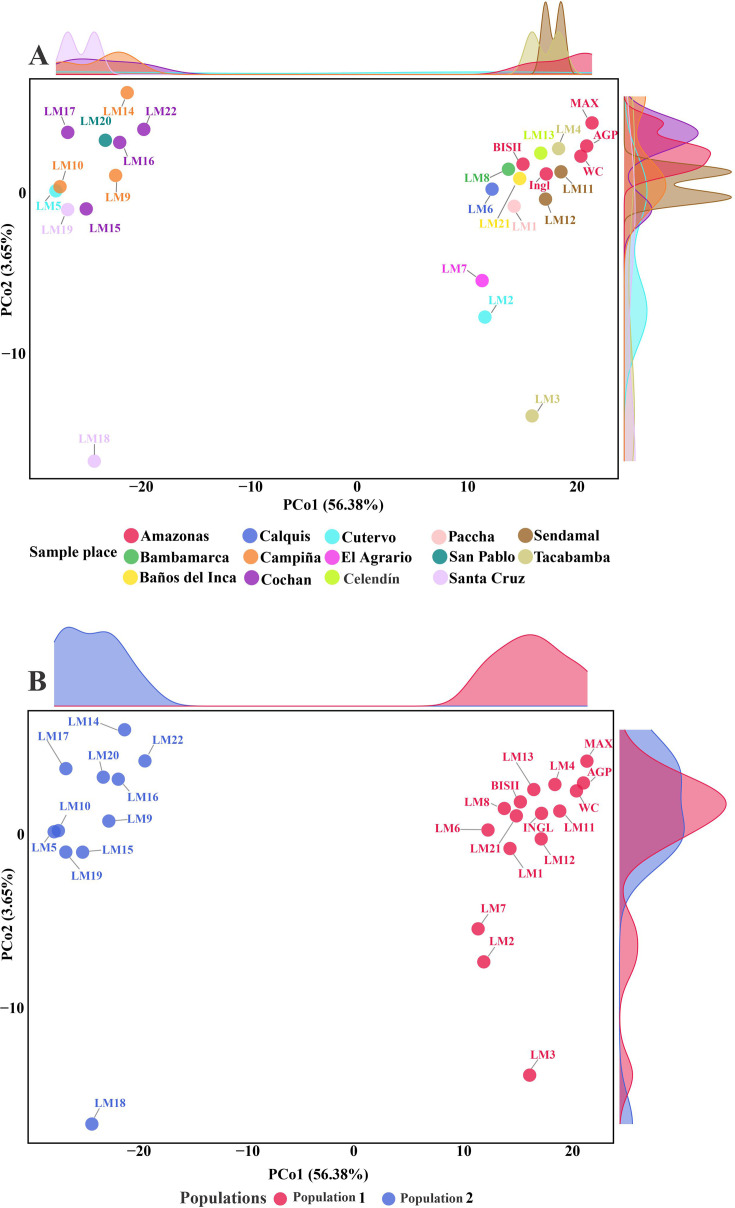
Principal coordinate analysis (PCoA) of 27 *L. multiflorum* accessions from the Peruvian highlands based on 2,070 SNPs. **(A)** The same PCoA colored by sampling location, illustrating the distribution of accessions across geographic origins and their correspondence with the inferred genetic groups. **(B)** PCoA colored according to the two main genetic groups (Population 1 and Population 2) inferred from the ADMIXTURE analysis at K = 2. The first two principal coordinates explain 56.38% (PCo1) and 3.65% (PCo2) of the total genetic variation, respectively.

Population structure among the 27 *L. multiflorum* accessions was evaluated using Principal Coordinate Analysis (PCoA), ADMIXTURE, hierarchical clustering, and genetic correlation analyses. The PCoA revealed consistent clustering patterns among accessions, with the first two principal coordinates explaining 56.38% and 3.65% of the total genetic variation, respectively (**Figure 2A**).

The ADMIXTURE analysis supported the presence of two genetically differentiated groups, with K = 2 identified as the most supported number of clusters based on the lowest cross-validation error (**Figure 2B**). The ancestry assignments showed a clear separation of the Cutervo accessions (LM5 and LM2), which were grouped into distinct clusters. In contrast, accessions from Cochán, Santa Cruz, Campiña, and San Pablo clustered within a single genetic group. Two accessions from Santa Cruz (LM18) and Tacabamba (LM13) displayed divergent positions relative to the main clusters. Accessions from Amazonas, Celendín, Sendamal, Baños del Inca, Paccha, Calquís, Bambamarca, and Tacabamba formed a consolidated genetic group. Additional divergence was observed for one accession from Tacabamba (LM3) and for accessions from El Agrario, which were positioned outside the main cluster.

**Figure 3:**
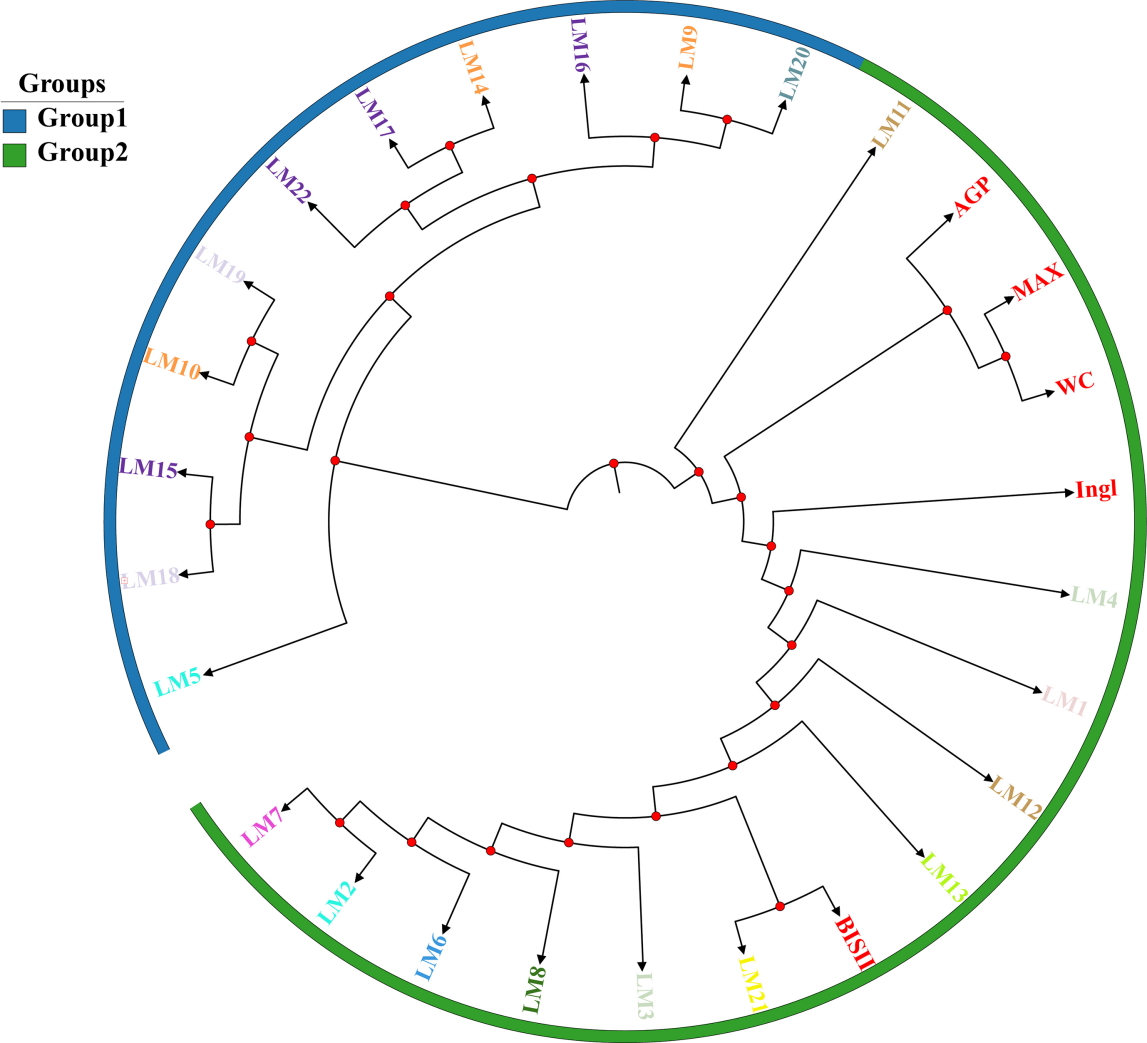
Phylogeny based on binary genetic distances and the unweighted pair group method with arithmetic mean (UPGMA) clustering method for the 27 ryegrass accessions using 2,070 single nucleotide polymorphism (SNP) markers. Detailed intragroup and intra-subgroup genetic distances are provided in **Supplementary Table 1**, whereas group and subgroup composition together with intra- and inter-genetic distances are summarized in **Supplementary Table 2**.

The phylogenetic tree reconstructed using the UPGMA method, based on binary distances from 2,070 high-quality SNPs (**Figure 3**), revealed a bifurcated structure composed of two main phylogenetic groups. The first group consists of 11 accessions from Cochan, Santa Cruz, Campiña, Cutervo, and San Pablo. The second group comprises 16 accessions from Amazonas, Tacabamba, Cutervo, El Agrario, Paccha, Sendamal, Baños del Inca, Calquis, Bambamarca, and Celendín. This phylogenetic structure is consistent with the PCoA results. To facilitate the interpretation of the phylogenetic structure, detailed information on intragroup and intra-subgroup genetic distances together with accession composition is provided in **Supplementary Table 1**. Additional information describing group and subgroup composition, as well as intra- and inter-genetic distances derived from the UPGMA analysis, is presented in **Supplementary Table 2**.

**Figure 4:**
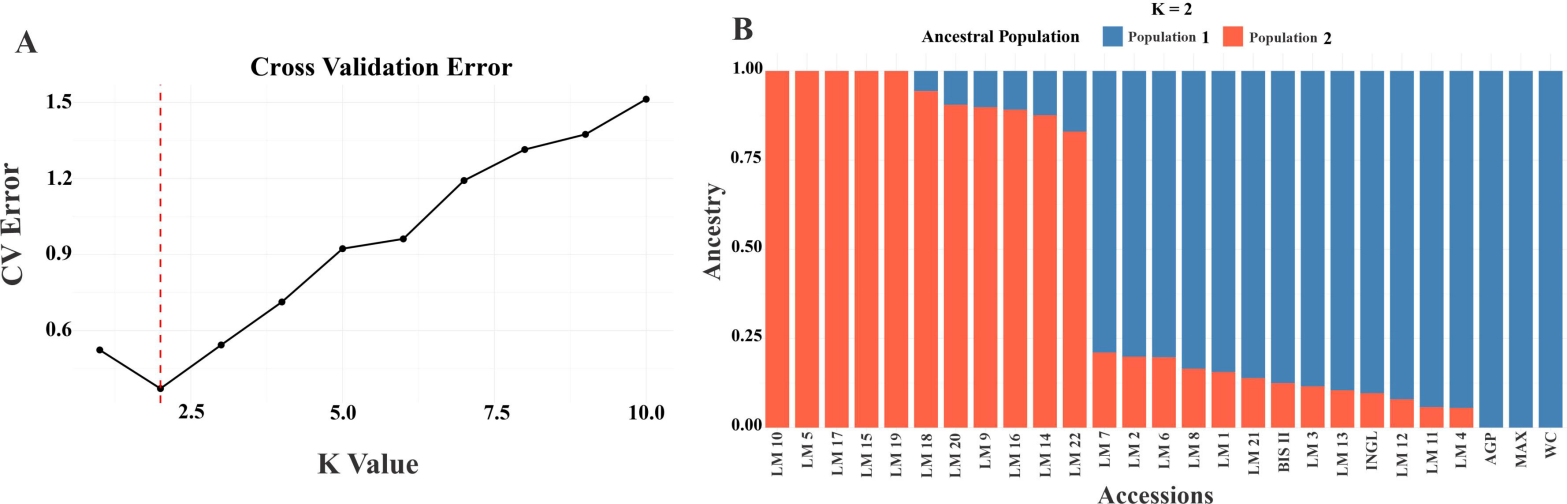
**(A)** Cross-validation error plot identifying K = 2 as the optimal number of ancestral populations. **(B)** Ancestry bar plot at K = 2 showing two genetically differentiated groups, with several individuals exhibiting mixed ancestry.

The genetic structure analysis performed with ADMIXTURE identified K = 2 as the optimal number of ancestral populations, determined by the lowest cross-validation error (**Figure 4A**). Under this model, the 27 accessions were clearly grouped into two genetically differentiated populations (**Figure 4B**). Population 1 (blue) included 16 accessions, while Population 2 (orange) comprised 11 accessions. Within Population 1, the accessions AGP, MAX, and WC formed a differentiated subgroup, whereas in Population 2, the accessions LM10, LM5, LM17, LM19, and LM15 constituted another clearly defined subgroup.

To evaluate the genetic differentiation between accessions with apparently pure ancestry from population 1 (AGP, MAX, and WC) and population 2 (LM10, LM5, LM17, LM15, and LM19) and those showing mixed ancestry within each population, Weir and Cockerham’s FST estimator (Weir and Cockerham, 1984) was calculated. In population 2, the FST between pure and admixed individuals was 0.0082, whereas in population 1 the value was 0.0216 (**Table 3**). Both values indicated low genetic differentiation within populations. In contrast, higher differentiation was observed between populations, with an overall FST value of 0.057.

**Table 3:** Weir and Cockerham FST matrix among the groups defined based on the ADMIXTURE results.

	Population 2 – pure	Population 2 – admixed	Population 1 – pure	Population 1 – admixed
Population 2 – Pure	0.0000	0.0082	0.4610	0.3377
Population 2 – Admixed	0.0082	0.0000	0.3850	0.2830
Population 1 – Pure	0.4610	0.3850	0.0000	0.0216
Population 1 – Admixed	0.3377	0.2830	0.0216	0.0000

**Figure 5:**
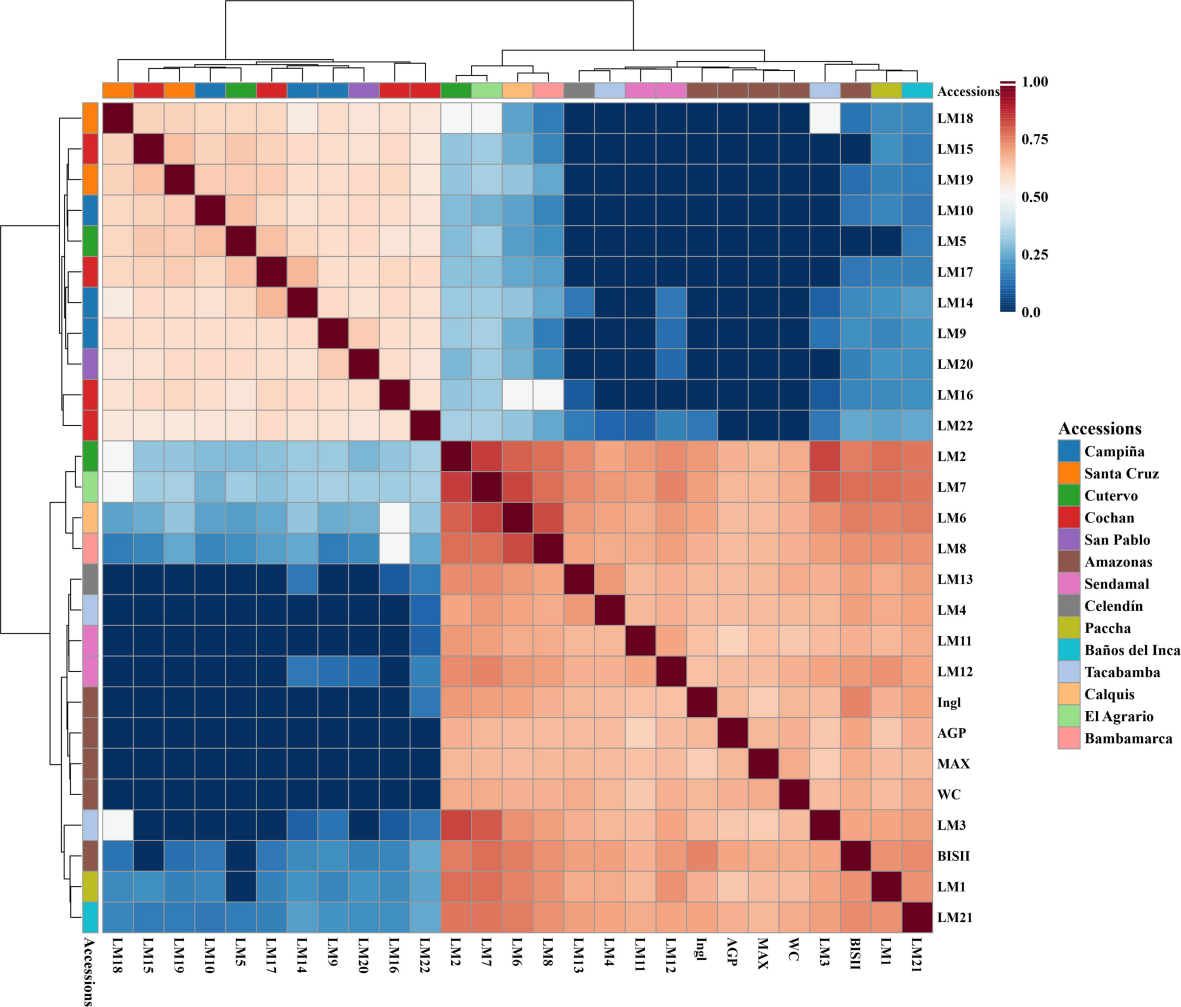
Heatmap of the individual-by-individual genetic correlation matrix based on SNPs.

The individual-by-individual genetic correlation matrix based on SNPs, together with hierarchical clustering, showed a clear segregation of individuals into two main groups (**Figure 5**). The first group was composed of individuals LM18, LM15, LM19, LM10, LM5, LM17, LM14, LM9, LM20, LM16, and LM22, which exhibited high genetic correlation values among themselves, as evidenced by warm color tones in the matrix. The second group included individuals LM2, LM7, LM6, LM8, LM13, LM4, LM11, LM12, AGP, MAX, WC, LM3, BISII, LM1, and LM21, likewise showing high levels of intragroup correlation. Correlations between individuals belonging to opposite groups were markedly lower, reflected by cool color tones. The main diagonal displayed maximum correlation values, corresponding to the genetic identity of each individual with itself (**Figure 5**).

### 3.3 Population structure and genetic differentiation

The AMOVA revealed significant genetic structure among the populations. A total of 10% of the genetic variation was attributed to differences among populations, whereas most of the diversity (90.01%) was found within populations. This population differentiation was statistically significant (PhiPT = 0.0999, p = 0.006), thus indicating that the groups analyzed are not genetically homogeneous and that processes such as drift, selection, or barriers to gene flow have effectively generated a moderate but meaningful genetic structure (**Table 4**). Additionally, statistical significance was evaluated using a randomization test with 999 permutations, which indicated that approximately 10% of the genetic variation can be explained by population structure (F = 2.776, p < 0.015) (**Supplementary Figure 2**).

**Table 4:** Analysis of molecular variance (AMOVA) of 27 *L. multiflorum* accessions based on single nucleotide polymorphism (SNP) markers.

Source of variation	DF	SSD	MSD	Variance	%	PhiPT	P-valor
Among populations*	1	2,121.1	2,121.10	81.55	9.99	0.0999	0.006
Within populations	25	19,102.8	764.11	734.75	90.01		
Total	26	21,223.9	816.30	816.30	100		

DF: degrees of freedom; SSD: sum of squares deviations; MSD: mean square deviations; pHiPT: genetic differentiation index.

*The population refers to the origin of the samples.

### 3.4 Genetic diversity patterns

The genetic diversity analysis based on 2,070 SNPs revealed moderate to high levels of genetic diversity in both populations. The F_IS_ values were negative, with -0.231 in Cajamarca and –0.549 in Amazonas. The observed heterozygosity (Ho) showed high values in Cajamarca (0.569) and Amazonas (0.664), while the expected heterozygosity (He) presented moderate levels in Cajamarca (0.463) and Amazonas (0.429).

Genetic diversity, assessed using the Shannon index, was higher in Cajamarca (0.857) compared to Amazonas (0.572) (**Table 5**).

**Table 5:** Genetic diversity between the Cajamarca and Amazonas populations of 27 ryegrass accessions.

Population	N	Ho	He	F_IS_	Shannon
Cajamarca	22	0.57	0.46	-0.2312	0.8568
Amazonas	5	0.66	0.43	-0.5489	0.5722

N: sample size; Ho: observed Heterozygosity; He: expected Heterozygosity; F_IS_: inbreeding coefficient (fixation Index).

The principal component analysis (PCA) based on productive traits (DM and FF) revealed a clear phenotypic differentiation among the accessions (**Supplementary Figure 3**). Commercial cultivars such as BISII, MAX, AGP, and INGL clustered in a region characterized by intermediate dry matter levels and low fresh forage production, whereas the remaining accessions were distributed across a broader range of phenotypic combinations.

## 4 Discussion

Genetic diversity is a key component of plant breeding, as it supports the selection and development of superior genotypes (Bhanu, 2017; Chauhan et al., 2025). In this context, molecular markers, particularly SNPs, have enabled a more precise characterization of genetic structure and variability within germplasm banks (Geethanjali et al., 2024). However, in outcrossing species, variation within cultivars is often high due to extensive pollen dispersal among them, which results in low levels of differentiation between populations (Roldán-Ruiz et al., 2000). These characteristic limits the effectiveness of traditional identification methods, thereby highlighting the need to employ high-throughput markers to optimize selection and accelerate the development of cultivars with superior agronomic traits and greater adaptability (Yu et al., 2022). To date, only limited research using molecular markers has been conducted to determine the genetic diversity of ryegrass in Peru. Here, for the first time, we employed genome-wide SNPs (**Figure 1**) to infer the genetic diversity and population structure of ryegrass germplasm from the northeastern highlands of Peru (**Table 1**).

A total of 2,070 high-quality SNPs were identified across the seven chromosomes of *L. multiflorum*, with SNIPs detected in both genic and intergenic regions, providing a valuable resource for characterizing genetic diversity and population structure (**Figure 2**). These results are similar to those reported by Blackmore et al. (2016), who examined the role of genetic diversity in *Lolium perenne* using 2,199 SNPs. According to the results of the analyses of the 27 ryegrass accessions, these can be divided into two main groups (**Figure 2**). Group I comprised fewer representatives and consisted of local accessions from Cochan, Campiña, Santa Cruz, San Pablo, and one accession from Cutervo, all originating from the Cajamarca Region. In contrast, Group II included a larger set, composed mainly of commercial accessions from the Amazonas Region such as MAX, BIS II, INGLES, and the AGPy Wanca Grass ecotype, which are closely related to materials from Sendamal, Celendín, Tacabamba, Bambamarca, Calqui, Paccha, Baños del Inca, and El Agrario, as well as an additional individual from Cutervo and Tacabamba.

The formation of these groups may be influenced by factors such as agricultural selection or environmental conditions (Xu et al., 2017). The geographic and climatic heterogeneity of Cajamarca could restrict gene flow and promote genetic divergence (Balfourier et al., 2019). Although Cajamarca and Amazonas are not separated by a strict geographical barrier, the variation in topography, altitude, and environmental conditions across the Andean landscape can contribute to genetic structuring while maintaining overall population connectivity. Interestingly, previous research in *L. multiflorum* Lam. reported a similar pattern of genetic structuring (Tamura et al., 2022), where the analysis of 456 SNPs distinguished two main groups: one composed of domesticated cultivars and landraces, and another corresponding to the introduced cultivar ‘Gulfo’. Consistent with this, our results showed that the commercial accessions from Amazonas and the cultivar LM22 (INIA910) share the same genetic group, thus suggesting a possible common ancestor. Notably, INIA910 exhibited a close relationship with the BISII cultivar from Amazonas, forming a monophyletic clade (**Figure 3**). This cultivar has been reported for its high productivity and nutritional content (Vásquez et al., 2025), which may explain its regional distribution.

The Cajamarca ecotype described by Vallejos-Cacho et al. (2024) and Rojas-Vásquez et al. (2023) was located within Group I, together with the cultivars from Campiñas, Cajamarca, and San Pablo, thereby forming a monophyletic clade (**Figure 3**). This pattern confirms a regional genetic differentiation, likely influenced by agricultural selection processes and by the geographic and climatic heterogeneity of Cajamarca (Balfourier et al., 2019). The presence of moderate gene flow among populations suggests that genetic connectivity persists, although with marked signals of local adaptation (Cheng et al., 2020). This ecotype is also characterized by its high nutritional content (Vallejos-Cacho et al., 2024). An important breeding application is to continue improving genetic gains within each population, e.g. through recurrent selection, while maintaining genetic distance between the two groups. This strategy enables the later exploitation of hybridization power by crossing progenitors from different groups, similar to approaches currently used in other crops such as maize (Xiao et al., 2021).

The genetic structure analysis using ADMIXTURE clustered the 27 ryegrass accessions from the Peruvian highlands into two well-defined genetic populations (**Figure 4**), suggesting differentiated groups potentially associated with local adaptation and environmental heterogeneity. This pattern is supported by the overall FST value of 0.057, indicating moderate but evident genetic differentiation between populations. In contrast, the FST values estimated between individuals with apparently pure and mixed ancestry within each population were low (0.0082 for Population 2 and 0.0216 for Population 1; **Table 3**), remaining below the threshold commonly associated with weak genetic differentiation (Hartl and Clark, 1997; Charlesworth, 1999). These results indicate limited internal differentiation and suggest that the ancestry patterns detected by ADMIXTURE are more likely related to natural variation within populations rather than strong internal subdivision. This interpretation is further supported by kinship relationships based on PI_HAT (**Figure 5**).

However, the presence of a third cluster (K = 3) indicates a finer genetic substructure that may reflect founder effects, genetic drift, or recent adaptation to local microenvironments (**Supplementary Figure 1**). This type of hierarchical structure is characteristic of allogamous species of the genus *Lolium*, where high gene flow coexists with the formation of genetically coherent groups associated with local selective pressures. Consistently, Kovi et al. (2015) reported K = 2 as the optimal number of populations *in L. perenne*, demonstrating that genetic differentiation can arise in response to specific selective pressures even in the absence of geographic isolation. Similarly, Nam et al. (2025) identified two main subpopulations (K = 2) in *Lolium* varieties analyzed with SSR markers, distinguishing diploid and tetraploid groups, and reported increased structural resolution when increasing to K = 3. In the present study, however, ploidy levels were not assessed; therefore, the finer substructure observed at K = 3 is more plausibly attributed to underlying genetic differentiation driven by local adaptation, demographic processes, or management-related factors rather than cytogenetic variation.

On the other hand, the integrated analysis of the productive principal components for dry matter (DM) and green forage (GF) (**Supplementary Figure 3**) showed that commercial accessions such as BISII, MAX, AGP, and INGL cluster in a region characterized by intermediate levels of dry matter and low green forage production, suggesting a more compact ideotype with lower aerial biomass volume but moderate productivity in terms of DM accumulation.

Genetically, individual-by-individual genetic correlation analyses (**Figure 5**), together with the population structure inference obtained from ADMIXTURE, indicate that these cultivars are consistently integrated within the same genetic population, a pattern that is reflected in their close proximity in the PCoA, indicating that they share a set of frequent alleles associated with this medium-to-low GF production profile (**Supplementary Figure 3**), consistent with improved materials selected for stability rather than maximum forage volume (Zhu et al., 2025a). In contrast, LM13, which in the PCA is located at the low end of DM and GF, also falls within this same genetic group in the PCoA but in a more peripheral position (**Supplementary Figure 3**), suggesting that although it shares ancestry with the commercial cultivars, it carries a less favorable allelic combination for forage productivity—possibly due to the loss of advantageous alleles or retention of variants associated with more conservative growth strategies (Verwimp et al., 2018; Nie et al., 2019). Meanwhile, ryegrass accessions L21 and L22 occupy an intermediate position in the PCA with moderate levels of DM and GF, and in ADMIXTURE they exhibit mixed ancestry with contributions from both ancestral genetic populations, which is consistent with their placement near the transition zone in the PCoA. This mixed genetic background may explain their intermediate phenotype and greater plasticity, acting as bridge genotypes between more productive lineages and others with lower yield (Faville et al., 2017; Fu and Wang, 2023).

Previous research in ryegrass indicates that its outcrossing nature and high hybridization capacity promote extensive genetic introgression among coexisting populations, which is facilitated by wind-driven pollen dispersal and seed exchange, thereby maintaining high genetic variability within populations (Beckie and Jasieniuk, 2021). Our results align with this pattern. The AMOVA showed that 90.1% of the total genetic variation is concentrated within populations, while only 9.99% is explained among populations (**Table 4**). This confirms that allelic diversity is predominantly shared at the intrapopulation level, and that differentiation among groups is low. This behavior has been widely documented in ryegrass ecotypes and cultivars (Vieira et al., 2004; Guthridge et al., 2001).

The genetic structure observed suggests high gene flow, likely associated with outcrossing reproduction, efficient pollen dispersal, and management practices that favor germplasm exchange (Guan et al., 2017; Nam et al., 2023). This is reinforced by the high observed heterozygosity values (Ho = 0.57–0.66), which consistently exceed the expected heterozygosity (He = 0.46–0.43) (**Table 5**). Although this value is lower than the 0.71 reported by Nam et al. (2025) for South Korea, it is similar to the values reported by Tamura et al. (2022) (0.562) and Blackmore et al. (2016) (0.33) for the United Kingdom. The higher values observed in the Peruvian highland populations suggest greater genetic mixing or environmental variability that maintains high levels of diversity (Castro et al., 2024).

Differences among these investigations may be attributed to the type of markers used. While simple sequence repeats (SSRs) are multiallelic and have high discriminatory power, SNPs are biallelic and require a larger number of polymorphic loci to achieve equivalent resolution to that offered by multiallelic SSR markers (Guichoux et al., 2011; Singh et al., 2013). Nevertheless, the consistency observed among investigations that use SNPs reinforces the reliability of the pattern detected in the present study.

Regarding the genetic diversity profile, contrasting characteristics were observed between populations. The notably negative F_IS_ values (–0.231 in Cajamarca and –0.549 in Amazonas) indicate a systematic excess of heterozygotes in both populations. These results align with those reported in European ecotypes (–0.06 to –0.11), thereby indicating an excess of heterozygotes associated with an outcrossing mating system (Blackmore et al., 2016). Similarly, research in Norway with *L. perenne* reported values of –0.039 (Kovi et al., 2015). However, the more negative values observed in the Peruvian highland populations suggest greater gene mixing or gene flow, possibly driven by germplasm exchange among communities, traditional agricultural practices, or the coexistence of multiple genetic materials in nearby areas (Zhu et al., 2025b). The observed heterozygote excess is biologically explained by the predominantly self-incompatible reproductive system of ryegrass, which operates through a gametophytic mechanism controlled by the *S* and *Z* loci, enforcing obligate outcrossing and maintaining high levels of heterozygosity within populations despite occasional breakdowns under specific environmental conditions (Cropano et al., 2021). The excess of heterozygotes is an expected characteristic in species with an obligatory outcrossing mating system (Blackmore et al., 2015). This pattern strongly suggests the presence of heterosis or balancing selection, in which heterozygous genotypes have an adaptive advantage (Wang et al., 2014). Although interspecific hybridization is a plausible hypothesis that could contribute to this pattern, the history of intensive breeding in ryegrass may have directly favored the retention of beneficial alleles in heterozygous condition (Labroo et al., 2021; Yanniccari et al., 2018; Karn and Jasieniuk, 2017; Knorst et al., 2018). The evidence of hybrid vigor and balancing selection indicates that breeding history and management practices have favored the retention of beneficial alleles in heterozygous form, which, combined with the high intrapopulation diversity, positions these germplasms as strategic resources for breeding programs, facilitating the introgression of desirable traits and the development of adapted cultivars. Moreover, this pattern underscores the importance of their conservation in the face of genetic drift and selective pressures arising from human management and intensive cultivation (Ghamkhar et al., 2023; Knorst et al., 2019; Ozkose and Tamkoc, 2014; Yang et al., 2023).

The determination and management of ploidy level constitute a strategic axis within *Lolium* breeding programs, considering that ryegrass naturally occurs in a diploid state (2n = 14) and also includes artificially induced tetraploids (4n = 28), which have been widely incorporated into modern forage systems due to their distinct agronomic attributes (Faville et al., 2022; Tozer et al., 2017). Diploid genotypes, characterized by smaller cell size and higher sward density, have traditionally been used in older cultivars and turf-specific applications, whereas tetraploids exhibit larger cells, greater palatability and nutritional value for livestock, as well as improved stress tolerance, although they generally show lower sward density and reduced long-term persistence (Guo et al., 2018; Pembleton et al., 2018).

In the present study, although this work provides the first genomic characterization of *L. multiflorum* germplasm from the Peruvian Andes, an important limitation is the lack of ploidy evaluation in the analyzed accessions. Karyotype studies, high-throughput sequencing (HTS), and chromosome conformation capture sequencing (Hi-C) have confirmed that *L. multiflorum* is predominantly diploid (2n = 14) (Abbaszade et al., 2017; Pašakinskienė et al., 1997; Knorst et al., 2018; Chen et al., 2025; Brunharo et al., 2025), although induced tetraploid cultivars (4n = 28) have also been reported (Ahloowalia, 1967; Rocha et al., 2018). In natural populations of the Peruvian Andes, the occurrence of tetraploids has not been systematically documented; however, the presence of individuals with different ploidy levels cannot be ruled out. Therefore, the population subdivision observed at K = 3 should be interpreted with caution, as it may partially reflect undetermined cytogenetic variation. Furthermore, future studies should incorporate broader geographic sampling and explicitly evaluate ploidy using cytogenetic approaches (Meirmans et al., 2018; Perez-Vicente et al., 1992). Such efforts would improve diversity estimates and provide stronger support for breeding programs and conservation strategies focused on Andean germplasm.

To exploit the genetic diversity identified in the present study, ryegrass breeding programs commonly use strategies such as polycross schemes and synthetic populations (Syn1), in which multiple elite progenitors are intercrossed to maintain high levels of heterozygosity associated with forage yield and digestibility (Faville et al., 2018, 2022; Wang et al., 2016). Similarly, half-sib family selection has enabled the identification of superior maternal lines for forage quality under controlled conditions (Wilkins, 1991; Cornish et al., 1979). In parallel, the use of doubled haploids generated through another culture have facilitated the development of homozygous materials required for breeding purposes (Begheyn et al., 2016).

In this context, the genomic diversity and population structure identified in the Peruvian *L. multiflorum* germplasm may provide useful information for future breeding programs, particularly for parent selection, conservation of locally adapted materials, and the identification of contrasting genetic backgrounds. The differentiation observed between populations, together with the high intrapopulation diversity and excess of heterozygosity, suggests the existence of valuable genetic resources that could be incorporated into improvement strategies. Beyond these conventional approaches, the application of genomic selection approaches based on GBS represents a promising future direction for ryegrass improvement. Previous studies have shown that genomic prediction can improve the selection of traits associated with dry matter yield, forage quality, persistence, disease resistance, and environmental adaptation in both diploid and tetraploid germplasm (Guo et al., 2018; Pembleton et al., 2018). Therefore, future studies integrating broader germplasm collections, phenotypic evaluations, association analyses, and genomic prediction approaches may strengthen breeding programs and accelerate cultivar development for Andean livestock systems.

Within this framework, the strategic management of germplasm with different ploidy levels, with particular emphasis on locally adapted diploid materials, emerges as a fundamental tool to enhance resilience to climatic variability while simultaneously optimizing forage productivity and quality of ryegrass in Andean livestock systems. Collectively, this work represents an initial genomic characterization of Peruvian *L. multiflorum* germplasm, laying the foundation for future studies that integrate broader collections, phenotypic assessments, and genomic prediction approaches to strengthen ryegrass breeding and conservation programs in Andean environments.

## 5 Conclusion

This study provides the first genomic assessment of genetic diversity of *L. multiflorum* accessions from the northern Peruvian highlands using genotype-by-sequencing. A total of 2,070 high-quality SNPs were identified, revealing the existence of two well-defined genetic populations. Cluster analysis and PCoA separated the accessions into two groups: a smaller group comprising the Cajamarca ecotype and local cultivars from Cochan, San Pablo, Campiña, Cutervo, and Santa Cruz; and a larger group including all commercial cultivars from Amazonas (Chachapoyas), along with local cultivars from Sendamal, Celendín, Bambamarca, Tacabamba, Calquis, Paccha, El Agrario, Cutervo, and the INIA 910 cultivar. These genetic relationships reflect a moderate yet significant population structure, alongside high genetic diversity within populations, particularly in Cajamarca. Collectively, these findings position Peruvian germplasm as a valuable resource for breeding programs aimed at improving forage productivity, nutritional quality, environmental adaptation, persistence, and stress tolerance. Following agronomic and phenotypic characterization, this genetic resource may support parent selection, genomic-assisted breeding strategies, and conservation efforts, thereby contributing to the sustainability and resilience of mountain livestock systems.

## Data Availability

The raw sequencing data generated in this study have been deposited in the NCBI BioProject database under accession number PRJNA1427078.
