# Supplementary material for: Genomic insights into Lolium multiflorum diversity for forage breeding in Andean livestock systems
Source: Front Plant Sci. 2026 Jun 10;17:1818688. doi: 10.3389/fpls.2026.1818688 (PMC13290733; doi:10.3389/fpls.2026.1818688)
Supplement: Supplementary file 1 [file DataSheet1.docx]

Supplementary Material

# Supplementary Data

# The supplementary material includes additional figures and tables supporting the genomic analyses of *Lolium multiflorum* accessions. These data provide complementary information on population structure, genetic diversity,

# Supplementary Figures and Tables

## Supplementary Figures


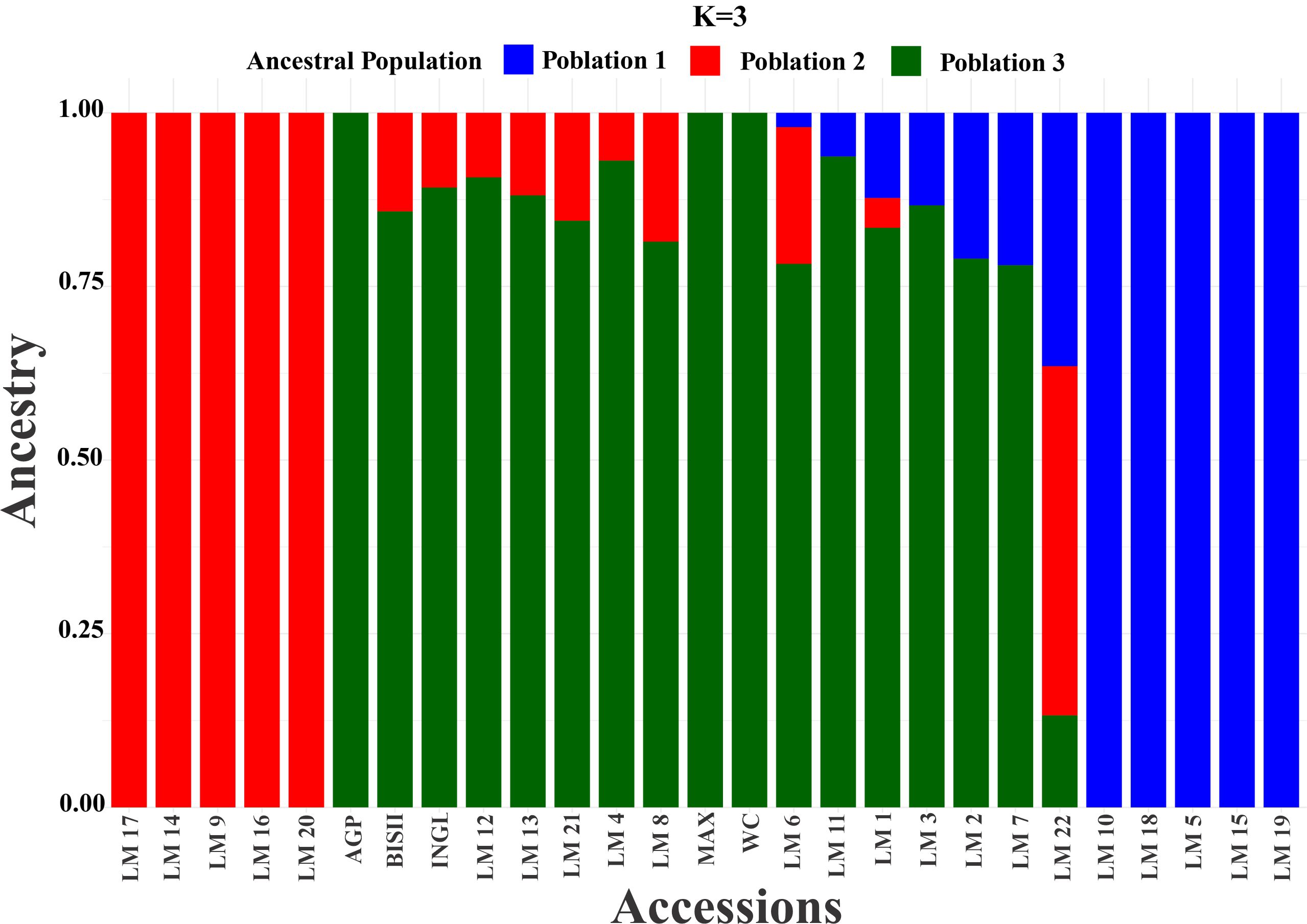


**Figure S1.** Genetic structure analysis at K = 3


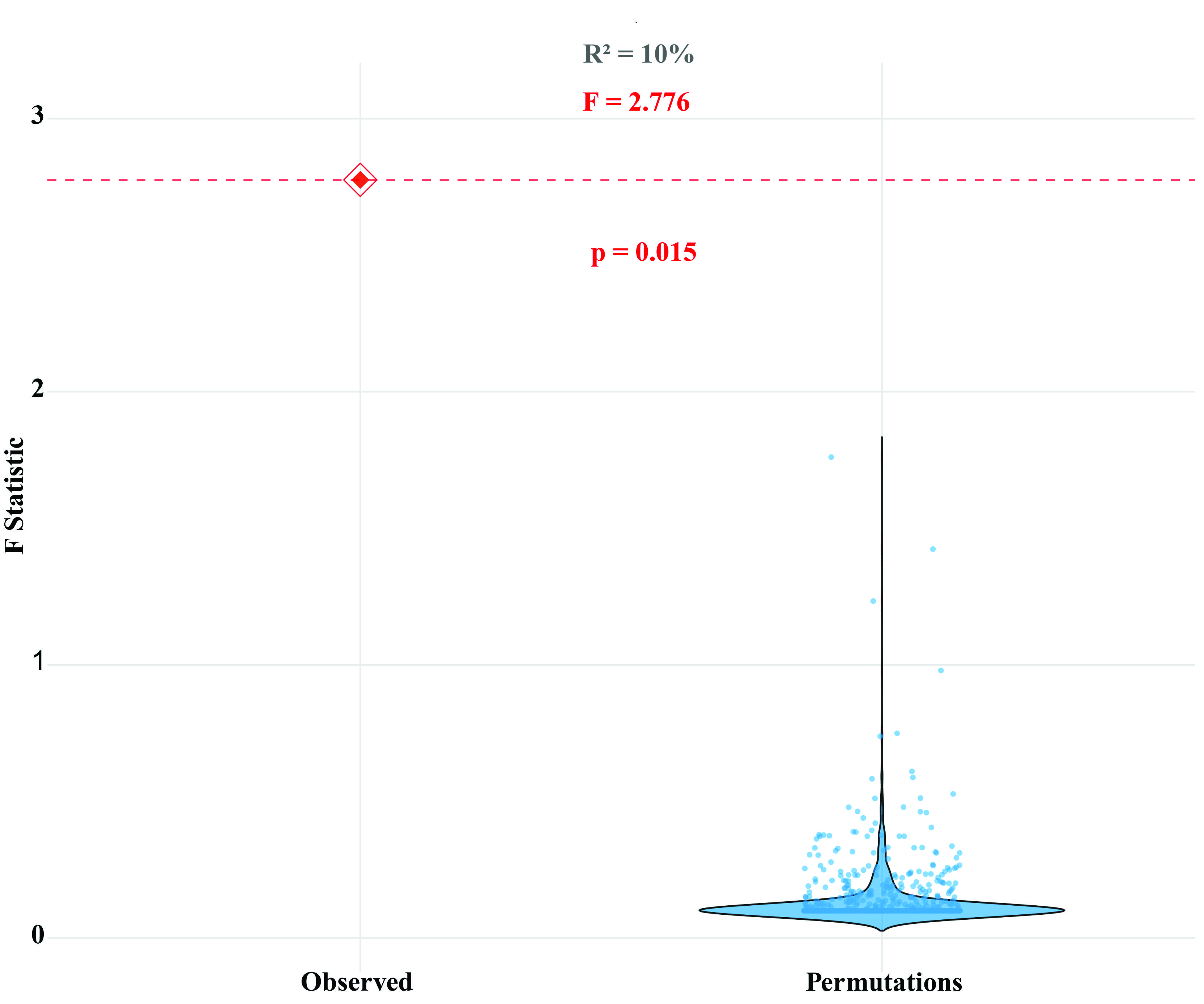


**Figure S2.** Distribution of permutations (n = 999). The red line indicates the observed F value of 2.776 (p = 0.015).


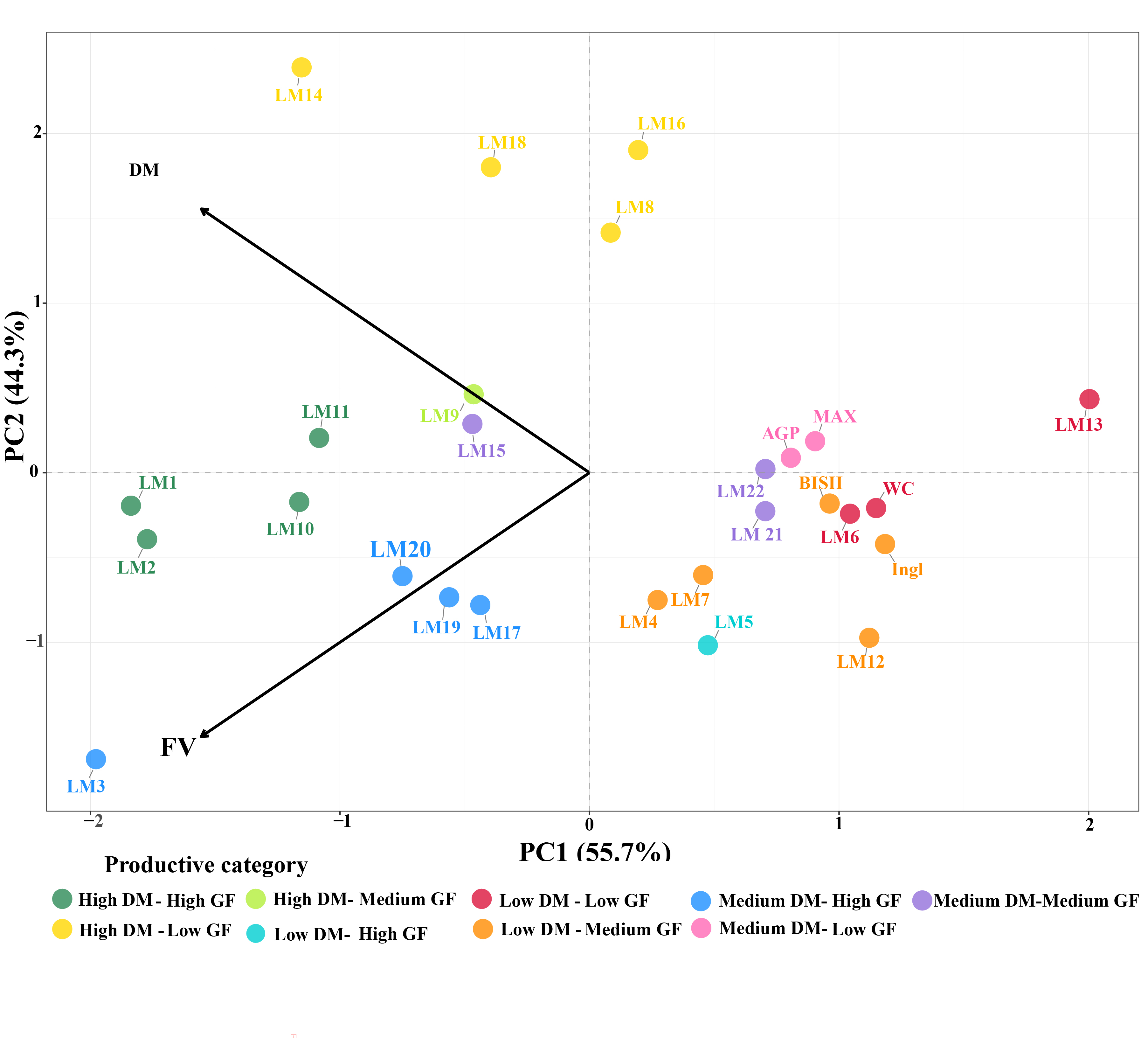


**Figure S3.** PCA analysis of 27 ryegrass accessions. The colors indicate combinations of dry matter (DM) and green forage (GF) across the different cultivars: dark green, cultivars with high DM and high GF; yellow, high DM and low GF; light green, high DM and medium GF; light blue, low DM and high GF; red, low DM and low GF; orange, low DM and medium GF; blue, medium DM and high GF; pink, medium DM and low GF; purple, medium DM and medium GF.

## Supplementary Tables

**Table S1.** Intragroup and intra-subgroup genetic distances and accession composition derived from UPGMA clustering analysis of 27 *Lolium multiflorum* accessions based on 2,070 SNP markers.

| **Intragroup distances** | | | |
| --- | --- | --- | --- |
| **Group** | **Samples** | **Distance_intra_mean** | **Distance_intra_std** |
| Amazonas | AGP, BISII, Ingl, MAX, WC | 0.66651988 | 0.009953374 |
| Campiña | LM10, LM14, LM9 | 0.987522 | 0.008450613 |
| Cochan | LM17, LM15, LM16, LM22 | 0.970334733 | 0.009796511 |
| Cutervo | LM5, LM2 | 1.5158845 | 0.005369036 |
| Santa Cruz | LM18, LM19 | 0.9628566 | 0.026889156 |
| Sendamal | LM11, LM12 | 0.7184623 | 0.014524855 |
| Tacabamba | LM3, LM4 | 0.6326583 | 0.013014575 |
| **Intra-subgroup distances** | | | |
| **Subgroup** | **Samples** | **Distance_intra_mean** | **Distance_intra_std** |
| Amazonas_north | AGP, BISII, Ingl | 0.680557267 | 0.013680379 |
| Amazonas_south | MAX, WC | 0.6132386 | 0.028470687 |
| LM_high | LM18, LM17, LM19, LM16, LM20, LM22, LM21 | 1.127639933 | 0.005244933 |
| LM_under | LM5, LM1, LM2, LM3, LM4, LM6, LM7, LM8 | 0.7717151 | 0.006399503 |
| LM_medium | LM10, LM14, LM15, LM9, LM11, LM12, LM13 | 1.247355267 | 0.003322195 |

**Table S2.** Group and subgroup composition together with intra- and inter-genetic distances derived from UPGMA analysis of 27 *Lolium multiflorum* accessions based on 2,070 SNP markers.

| **Intergroup distances** | | | | | |
| --- | --- | --- | --- | --- | --- |
| **Group 1** | **Samples_Group 1** | **Group 2** | **Samples_Group 2** | **Distance_inter_mean** | **Distance_inter_std** |
| Amazonas | AGP, BISII, Ingl, MAX, WC | Bambamarca | LM8 | 0.6429278 | 0.008397098 |
| Amazonas | AGP, BISII, Ingl, MAX, WC | Baños del Inca | LM21 | 0.6429322 | 0.008346303 |
| Amazonas | AGP, BISII, Ingl, MAX, WC | Calquis | LM6 | 0.64290464 | 0.008429308 |
| Amazonas | AGP, BISII, Ingl, MAX, WC | Campiña | LM10, LM14, LM9 | 1.51588776 | 0.005365311 |
| Amazonas | AGP, BISII, Ingl, MAX, WC | Cochan | LM17, LM15, LM16, LM22 | 1.51588636 | 0.005366466 |
| Amazonas | AGP, BISII, Ingl, MAX, WC | Cutervo | LM5, LM2 | 1.07939169 | 0.004850983 |
| Amazonas | AGP, BISII, Ingl, MAX, WC | El Agrario | LM7 | 0.64288796 | 0.008437667 |
| Amazonas | AGP, BISII, Ingl, MAX, WC | Micuypampa | LM13 | 0.66300962 | 0.00855252 |
| Amazonas | AGP, BISII, Ingl, MAX, WC | Paccha | LM1 | 0.65484 | 0.008039479 |
| Amazonas | AGP, BISII, Ingl, MAX, WC | San Pablo | LM20 | 1.51588636 | 0.005367133 |
| Amazonas | AGP, BISII, Ingl, MAX, WC | Santa Cruz | LM18, LM19 | 1.51588696 | 0.00536747 |
| Amazonas | AGP, BISII, Ingl, MAX, WC | Sendamal | LM11, LM12 | 0.68556351 | 0.008716327 |
| Amazonas | AGP, BISII, Ingl, MAX, WC | Tacabamba | LM3, LM4 | 0.65383595 | 0.007814274 |
| Bambamarca | LM8 | Baños del Inca | LM21 | 0.513084 | 0.014301908 |
| Bambamarca | LM8 | Calquis | LM6 | 0.361817 | 0.031058384 |
| Bambamarca | LM8 | Campiña | LM10, LM14, LM9 | 1.5158834 | 0.00536986 |
| Bambamarca | LM8 | Cochan | LM17, LM15, LM16, LM22 | 1.515882 | 0.005371015 |
| Bambamarca | LM8 | Cutervo | LM5, LM2 | 0.99784615 | 0.008274807 |
| Bambamarca | LM8 | El Agrario | LM7 | 0.47835 | 0.019720252 |
| Bambamarca | LM8 | Micuypampa | LM13 | 0.6267073 | 0.015507811 |
| Bambamarca | LM8 | Paccha | LM1 | 0.5843702 | 0.016143777 |
| Bambamarca | LM8 | San Pablo | LM20 | 1.515882 | 0.005371679 |
| Bambamarca | LM8 | Santa Cruz | LM18, LM19 | 1.5158826 | 0.005371991 |
| Bambamarca | LM8 | Sendamal | LM11, LM12 | 0.64512655 | 0.011630079 |
| Bambamarca | LM8 | Tacabamba | LM3, LM4 | 0.55723105 | 0.010635296 |
| Baños del Inca | LM21 | Calquis | LM6 | 0.5129284 | 0.014593557 |
| Baños del Inca | LM21 | Campiña | LM10, LM14, LM9 | 1.515885 | 0.005368172 |
| Baños del Inca | LM21 | Cochan | LM17, LM15, LM16, LM22 | 1.5158836 | 0.005369322 |
| Baños del Inca | LM21 | Cutervo | LM5, LM2 | 1.01409975 | 0.007983459 |
| Baños del Inca | LM21 | El Agrario | LM7 | 0.5122876 | 0.015340421 |
| Baños del Inca | LM21 | Micuypampa | LM13 | 0.6267089 | 0.015510546 |
| Baños del Inca | LM21 | Paccha | LM1 | 0.584396 | 0.01607786 |
| Baños del Inca | LM21 | San Pablo | LM20 | 1.5158836 | 0.00536999 |
| Baños del Inca | LM21 | Santa Cruz | LM18, LM19 | 1.5158842 | 0.005370318 |
| Baños del Inca | LM21 | Sendamal | LM11, LM12 | 0.64515165 | 0.011614692 |
| Baños del Inca | LM21 | Tacabamba | LM3, LM4 | 0.57275545 | 0.010494737 |
| Calquis | LM6 | Campiña | LM10, LM14, LM9 | 1.515883 | 0.005370008 |
| Calquis | LM6 | Cochan | LM17, LM15, LM16, LM22 | 1.5158816 | 0.005371167 |
| Calquis | LM6 | Cutervo | LM5, LM2 | 0.99501595 | 0.01576503 |
| Calquis | LM6 | El Agrario | LM7 | 0.4701702 | 0.040372318 |
| Calquis | LM6 | Micuypampa | LM13 | 0.6267069 | 0.015507428 |
| Calquis | LM6 | Paccha | LM1 | 0.5843698 | 0.016143987 |
| Calquis | LM6 | San Pablo | LM20 | 1.5158816 | 0.005371825 |
| Calquis | LM6 | Santa Cruz | LM18, LM19 | 1.5158822 | 0.005372143 |
| Calquis | LM6 | Sendamal | LM11, LM12 | 0.64512615 | 0.011630943 |
| Calquis | LM6 | Tacabamba | LM3, LM4 | 0.55636165 | 0.011618831 |
| Campiña | LM10, LM14, LM9 | Cochan | LM17, LM15, LM16, LM22 | 0.964014667 | 0.007338847 |
| Campiña | LM10, LM14, LM9 | Cutervo | LM5, LM2 | 1.257502283 | 0.006520436 |
| Campiña | LM10, LM14, LM9 | El Agrario | LM7 | 1.5158842 | 0.005367017 |
| Campiña | LM10, LM14, LM9 | Micuypampa | LM13 | 1.5158873 | 0.005366512 |
| Campiña | LM10, LM14, LM9 | Paccha | LM1 | 1.5158858 | 0.005367596 |
| Campiña | LM10, LM14, LM9 | San Pablo | LM20 | 0.948860467 | 0.010842113 |
| Campiña | LM10, LM14, LM9 | Santa Cruz | LM18, LM19 | 0.989512633 | 0.009112546 |
| Campiña | LM10, LM14, LM9 | Sendamal | LM11, LM12 | 1.51588715 | 0.005367147 |
| Campiña | LM10, LM14, LM9 | Tacabamba | LM3, LM4 | 1.51588625 | 0.005366217 |
| Cochan | LM17, LM15, LM16, LM22 | Cutervo | LM5, LM2 | 1.258936225 | 0.0060817 |
| Cochan | LM17, LM15, LM16, LM22 | El Agrario | LM7 | 1.5158828 | 0.005368181 |
| Cochan | LM17, LM15, LM16, LM22 | Micuypampa | LM13 | 1.5158859 | 0.00536767 |
| Cochan | LM17, LM15, LM16, LM22 | Paccha | LM1 | 1.5158844 | 0.005368744 |
| Cochan | LM17, LM15, LM16, LM22 | San Pablo | LM20 | 0.9660106 | 0.0106462 |
| Cochan | LM17, LM15, LM16, LM22 | Santa Cruz | LM18, LM19 | 0.9838783 | 0.009622553 |
| Cochan | LM17, LM15, LM16, LM22 | Sendamal | LM11, LM12 | 1.51588575 | 0.005368313 |
| Cochan | LM17, LM15, LM16, LM22 | Tacabamba | LM3, LM4 | 1.51588485 | 0.005367378 |
| Cutervo | LM5, LM2 | El Agrario | LM7 | 0.91150445 | 0.015238105 |
| Cutervo | LM5, LM2 | Micuypampa | LM13 | 1.07129785 | 0.008103937 |
| Cutervo | LM5, LM2 | Paccha | LM1 | 1.05012855 | 0.008389793 |
| Cutervo | LM5, LM2 | San Pablo | LM20 | 1.25965985 | 0.005583917 |
| Cutervo | LM5, LM2 | Santa Cruz | LM18, LM19 | 1.2578446 | 0.007488679 |
| Cutervo | LM5, LM2 | Sendamal | LM11, LM12 | 1.0805074 | 0.006131165 |
| Cutervo | LM5, LM2 | Tacabamba | LM3, LM4 | 1.00883925 | 0.008478145 |
| El Agrario | LM7 | Micuypampa | LM13 | 0.6267081 | 0.01550991 |
| El Agrario | LM7 | Paccha | LM1 | 0.584371 | 0.016144503 |
| El Agrario | LM7 | San Pablo | LM20 | 1.5158828 | 0.005368848 |
| El Agrario | LM7 | Santa Cruz | LM18, LM19 | 1.5158834 | 0.005369207 |
| El Agrario | LM7 | Sendamal | LM11, LM12 | 0.64512735 | 0.011628755 |
| El Agrario | LM7 | Tacabamba | LM3, LM4 | 0.50357555 | 0.017712782 |
| Micuypampa | LM13 | Paccha | LM1 | 0.6279177 | 0.013449656 |
| Micuypampa | LM13 | San Pablo | LM20 | 1.5158859 | 0.005368325 |
| Micuypampa | LM13 | Santa Cruz | LM18, LM19 | 1.5158865 | 0.005368679 |
| Micuypampa | LM13 | Sendamal | LM11, LM12 | 0.67280745 | 0.010653953 |
| Micuypampa | LM13 | Tacabamba | LM3, LM4 | 0.61077365 | 0.014501447 |
| Paccha | LM1 | San Pablo | LM20 | 1.5158844 | 0.0053694 |
| Paccha | LM1 | Santa Cruz | LM18, LM19 | 1.515885 | 0.005369753 |
| Paccha | LM1 | Sendamal | LM11, LM12 | 0.65307965 | 0.011064869 |
| Paccha | LM1 | Tacabamba | LM3, LM4 | 0.60867405 | 0.011275661 |
| San Pablo | LM20 | Santa Cruz | LM18, LM19 | 0.9881599 | 0.01209498 |
| San Pablo | LM20 | Sendamal | LM11, LM12 | 1.51588575 | 0.005368953 |
| San Pablo | LM20 | Tacabamba | LM3, LM4 | 1.51588485 | 0.005368037 |
| Santa Cruz | LM18, LM19 | Sendamal | LM11, LM12 | 1.51588635 | 0.005369324 |
| Santa Cruz | LM18, LM19 | Tacabamba | LM3, LM4 | 1.51588545 | 0.005368382 |
| Sendamal | LM11, LM12 | Tacabamba | LM3, LM4 | 0.66034885 | 0.010065907 |
| **Inter-Subgroup distances** | | | | | |
| **Subgroup 1** | **Samples_Subgroup 1** | **Subgroup 2** | **Samples_Subgroup 2** | **Distance_inter_mean** | **Distance_inter_std** |
| Amazonas_north | AGP, BISII, Ingl | Amazonas_sur | MAX, WC | 0.6683814 | 0.009905313 |
| Amazonas_north | AGP, BISII, Ingl | LM_high | LM18, LM17, LM19, LM16, LM20, LM22, LM21 | 1.388929395 | 0.004742522 |
| Amazonas_north | AGP, BISII, Ingl | LM_under | LM5, LM1, LM2, LM3, LM4, LM6, LM7, LM8 | 0.745237842 | 0.007787772 |
| Amazonas_north | AGP, BISII, Ingl | LM_medium | LM10, LM14, LM15, LM9, LM11, LM12, LM13 | 1.155187043 | 0.004352467 |
| Amazonas_south | MAX, WC | LM_high | LM18, LM17, LM19, LM16, LM20, LM22, LM21 | 1.394552643 | 0.004794843 |
| Amazonas_south | MAX, WC | LM_under | LM5, LM1, LM2, LM3, LM4, LM6, LM7, LM8 | 0.772773138 | 0.009690623 |
| Amazonas_south | MAX, WC | LM_medium | LM10, LM14, LM15, LM9, LM11, LM12, LM13 | 1.1592505 | 0.005023078 |
| LM_high | LM18, LM17, LM19, LM16, LM20, LM22, LM21 | LM_under | LM5, LM1, LM2, LM3, LM4, LM6, LM7, LM8 | 1.338855646 | 0.004083772 |
| LM_high | LM18, LM17, LM19, LM16, LM20, LM22, LM21 | LM_medium | LM10, LM14, LM15, LM9, LM11, LM12, LM13 | 1.19494438 | 0.003053349 |
| LM_under | LM5, LM1, LM2, LM3, LM4, LM6, LM7, LM8 | LM_medium | LM10, LM14, LM15, LM9, LM11, LM12, LM13 | 1.150951011 | 0.004172061 |
